# Synkopenabklärung bei Kindern und Jugendlichen – Handeln wir gemäß der aktuellen Leitlinie?

**DOI:** 10.1007/s10354-020-00798-3

**Published:** 2021-01-13

**Authors:** Katharina Landwehr, Sascha Meyer, Marina Flotats-Bastardas, Martin Poryo

**Affiliations:** 1 Klinik für Allgemeine Pädiatrie und Neonatologie, Universitätsklinikum des Saarlandes, Homburg/Saar, Deutschland; 2 Klinik für Kinder und Jugendliche, Klinikum Leverkusen, Leverkusen, Deutschland; 3 Klinik für Allgemeine Pädiatrie und Neonatologie, Sektion Neuropädiatrie, Universitätsklinikum des Saarlandes, Homburg/Saar, Deutschland; 4 Klinik für Pädiatrische Kardiologie, Universitätsklinikum des Saarlandes, Homburg/Saar, Deutschland

**Keywords:** Synkope, Kinder und Jugendliche, Basisdiagnostik, Red flags, Leitlinie, Syncope, Children and adolscents, Basic diagnostic testing, Red flags, Guideline

## Abstract

**Hintergrund:**

Synkopen im Kindes‑/Jugendalter sind häufig und meist gutartig. Mögliche kardiale Synkopen müssen durch sorgfältige Basisdiagnostik (Anamnese (I), körperliche Untersuchung (II), Elektrokardiografie (III)) und ggf. weiterführender Diagnostik ausgeschlossen werden.

**Fragestellung:**

Wurde die Diagnostik bei Vorliegen einer Synkope entsprechend der gültigen S2k-Leitlinie durchgeführt?

**Material und Methoden:**

Retrospektive Analyse (01/2015–12/2017), Kinderklinik des Universitätsklinikums des Saarlandes, Homburg, Deutschland. Eingeschlossen wurden alle Patienten von 1 bis 18 Jahre, die sich wegen Synkope vorstellten.

**Ergebnisse:**

Es erlitten 262 Patienten eine Synkope (161 weiblich [61,5 %], 101 männlich [38,5 %], Alter 12,5 ± 3,9 Jahre); davon 183 (69,8 %) Reflexsynkopen, 36 (13,7 %) Präsynkopen, 35 (13,4 %) Synkopen unklarer Genese, 8 (3,1 %) kardiale Synkopen; 43/262 Patienten (16,4 %) erhielten eine vollständiger Basisdiagnostik (I–III) gemäß Leitlinie, 13/43 (30,2 %) wurden korrekt weiterführender Diagnostik zugeführt; 219/262 Patienten (83,6 %) erhielten keine ausreichende Basisdiagnostik (I–III), 135/219 (61,6 %) wurden unnötigen apparativen Untersuchungen zugeführt.

**Diskussion:**

Die leitlinienkonforme Synkopenabklärung ist wichtig, um unnötige, aber auch nicht ausreichende Diagnostik zu vermeiden und somit Patienten mit Synkope korrekt zu diagnostizieren.

## Summary

**Background:**

Syncope in childhood and adolescence is frequent and in most cases benign. A thorough history taking, complete physical examination, electrocardiography and further diagnostic work-up as indicated should rule out possible cardiac syncope.

**Objective:**

To evaluate whether the diagnosis of syncope was performed according to the currently valid S2k guideline.

**Material and methods:**

Retrospective study (January 2015–December 2017), University Children’s Hospital of Saarland, Homburg, Germany. All patients aged 1–18 years presenting with the primary complaint of syncope were included.

**Results:**

In this study 262 patients presented with a history of syncope (161 female (61.5%), 101 male (38.5%), median age 12.5 ± 3.9 years). Of these, 183 (69.8%) were reflex syncopes, 36 (13.7%) presyncopes, 35 (13.4%) undefined and 8 (3.1%) cardiac syncope. Out of 262 patients, 43 (16.4%) were diagnosed in accordance with the published guidelines and 13/43 (30.2%) correctly received further diagnostic work-up. In 219/262 patients (83.6%) basic diagnostic testing was not sufficient and 135/219 (61.6%) were submitted to further unnecessary diagnostic tests.

**Conclusion:**

Better adherence to the syncope guidelines bears the potential to avoid unnecessary and costly auxiliary medical tests while correctly diagnosing patients with syncope.

Eine Synkope beschreibt einen kurzzeitigen und vollständig reversiblen Bewusstseinsverlust durch eine zerebrale Minderperfusion [8]. Die häufigste Ursache hierfür ist die gutartige Reflexsynkope. In seltenen Fällen kann der Synkope allerdings eine kardiale Erkrankung zugrunde liegen, die einer weiteren Abklärung bedarf. Hierzu wurde kürzlich eine aktualisierte S2k-Leitlinie zur „Synkope im Kindes- und Jugendalter“ von der Deutschen Gesellschaft für Pädiatrische Kardiologie und Angeborene Herzfehler e. V. veröffentlicht (DGPK) [7].

Es wird davon ausgegangen, dass ungefähr jeder zweite Mensch im Laufe seines Lebens [2] und ca. jedes vierte Kind bis zum Erreichen des Erwachsenenalters mindestens eine Synkope [21] erleidet; das Rezidivrisiko beträgt dabei ca. 33–51 % [23]. Gutartige Reflexsynkopen treten dabei im Kindes- und Jugendalter deutlich häufiger auf als kardial bedingte Synkopen (ca. 2–6 % der pädiatrischen Fälle) [4, 18, 32]. Kardial bedingte Synkopen gehen sowohl im Erwachsenen- als auch im Kindes- und Jugendalter mit einer erhöhten Mortalität einher [18, 30]. Die häufigste und wichtigste Differenzialdiagnose der Synkope im Kindes- und Jugendalter ist der epileptische Anfall [24].

Aufgrund der Häufigkeit von Synkopen ist die Versorgung und Abklärung dieser Patienten mit relevanten Kosten im Gesundheitssystem verbunden [17]. Nicht zu vernachlässigen sind dabei die Gesundheits- und Sozialkosten, die durch nicht notwendige Diagnostik und Krankenhausaufenthalte entstehen [11, 29]; insbesondere durch neurologische und kardiologische Untersuchungen [29]. Bisherige Studien bei pädiatrischen Patienten mit Synkope zeigten eine positive Auswirkung der Umsetzung von Leitlinien [16, 26, 28]. So konnte in einer italienischen Studie durch die praktische Anwendung nicht nur die Diagnosestellung verbessert werden, sondern es kam gleichzeitig zu einem verminderten Einsatz nicht notwendiger diagnostischer Untersuchungen [28].

Das Ziel dieser retrospektiven Studie war es, das diagnostische Vorgehen bei Vorliegen einer Synkope in unserer pädiatrischen Patientenkohorte zu beschreiben und es mit der zum Zeitpunkt des Auftretens der Synkope gültigen S2k-Leitlinie der DGKP [8] in Bezug zu setzen.

## Patienten und Methode

Bei dieser Arbeit handelt es sich um eine retrospektive Datenerhebung (01/2015 bis 12/2017), die an der Kinderklinik des Universitätsklinikums des Saarlandes (UKS, Homburg/Saar, Deutschland) durchgeführt wurde. Die Durchführung der Studie wurde von der Ethik-Kommission der Ärztekammer des Saarlandes, Saarbrücken, Deutschland (Kenn-Nr. 247/17), bewilligt.

Es wurden alle Patienten eingeschlossen, die sich im Erhebungszeitraum mit erstmaliger, einmaliger oder rezidivierender Synkope vorstellten. Erfasst wurden sowohl Vorstellung über die Notaufnahme der Kinderklinik als auch über die kinderkardiologische und neurologische Spezialambulanzen der Kinderklinik am UKS. Hierzu wurden alle Fälle mit der ICD-10-Kodierung „R55: Synkope und Kollaps“ aus dem Krankenhausinformationssystem (SAP, Walldorf, Deutschland) abgefragt.

Ausgeschlossen wurden Patienten, die bereits vor dem genannten Erhebungszeitraum ambulant oder stationär wegen Synkope behandelt wurden, alle Patienten ohne Synkope als primärer Behandlungsgrund sowie Patienten ohne ausreichende Datendokumentation. Patienten mit Differenzialdiagnosen des vorübergehenden Bewusstseinsverlustes wurden identifiziert (neurologisch bedingter vorübergehender Bewusstseinsverlust, Intoxikation, psychogene Pseudosynkope, metabolisch bedingter vorübergehender Bewusstseinsverlust) und ebenfalls von der Datenauswertung ausgeschlossen.

Erhoben wurden relevante demografische Parameter des Patientenkollektivs (Alter, Geschlecht etc.), die Synkopenentität (Reflexsynkope, Präsynkope, kardiale Synkope und Synkope unklarer Genese), anamnestische Daten (Vorerkrankungen, Medikamenteneinnahme, Familienanamnese, Prodromi, Trigger und „red flags“) sowie Informationen zum Krankenhausaufenthalt. Als „red flags“ galten: Synkope im Liegen, Synkope während körperlicher Belastung, Synkope nach Brustschmerz oder Palpitationen, Synkope ohne Prodromi. Darüber hinaus wurden Informationen über die körperliche Untersuchung sowie die durchgeführte apparative Diagnostik (bestehend aus 12-Kanal-Elektrokardiogramm [EKG], transthorakaler Echokardiographie [TTE], Elektroenzephalographie [EEG], Laboruntersuchung des Blutes, Schellong-Test, Langzeitelektrokardiogramm [LZ-EKG], Langzeitblutdruckmessung [LZ-RR], Ergometrie, kranielle Magnetresonanztomographie [cMRT] und Blutzuckertagesprofil [BZTP]) erfasst.

Die Einteilung der Synkopen erfolgte gemäß der zum Zeitpunkt der Erhebung gültigen S2k-Leitlinie: Synkope im Kindes- und Jugendalter [8] in Reflexsynkopen, Synkopen infolge orthostatischer Hypotonie und kardiogene Synkopen.

Zu unterscheiden ist die Präsynkope von einer Synkope, bei der es zu Prodromi einer Synkope ohne einhergehenden Bewusstseinsverlust kommt [10, 17]. Aufgrund der gleichen Pathophysiologie sollten Präsynkope und Synkope einheitlich diagnostisch abgeklärt werden [3, 13] und wurden daher mit in unsere Auswertung einbezogen.

Abschließend wurde überprüft, ob die Patienten gemäß der S2k-Leitlinie „Synkope im Kindes- und Jugendalter“ diagnostiziert wurden [8]. Die Basisdiagnostik wurde entsprechend definiert als vollständige Anamnese (Basisdiagnostik I), körperliche Untersuchung inklusive Ruheblutdruckmessung (Basisdiagnostik II) sowie der Ableitung eines 12-Kanal-EKGs (Basisdiagnostik III). Zudem wurde untersucht, ob der Einsatz weiterführender Diagnostik wie TTE, EEG und/oder LZ-EKG indiziert war. Als Kriterien für den indizierten Einsatz einer weiterführenden Diagnostik galten gemäß [8]: anamnestisch das Vorliegen einer relevanten, insbesondere einer kardialen Vorerkrankung und das Vorliegen von „red flags“, ein auffälliger, insbesondere pathologischer kardiologischer Untersuchungsbefund, ein auffälliger Befund im 12-Kanal-EKG, ein Alter <10 Jahren oder andere anamnestische Auffälligkeiten.

In einem nächsten Schritt wurden unterdiagnostizierte von überdiagnostizierten Patienten differenziert. Ohne vollständig durchgeführte Basisdiagnostik I–III galten die Patienten als unterdiagnostiziert. Patienten, bei denen die Basisdiagnostik I–III ausreichend gewesen wäre und die weiteren Untersuchungen (TTE, EEG, LZ-EKG) zugeführt wurden, galten hingegen als überdiagnostiziert.

### Statistik

Die erhobenen Daten wurden pseudonymisiert und mithilfe des Programms IBM SPSS Statistics Version 24 (IBM Corp. Released 2016. IBM SPSS Statistics for Windows, Version 24.0. Armonk, NY: IBM Corp., USA) erfasst und ausgewertet. Die Ergebnisse sind als absolute (*n*) und relative Häufigkeiten (%), Mittelwert (MW), Standardabweichung (SD) und Spannweite angegeben. Zur statistischen Testung wurde der *Chi*^*2*^*-Test* durchgeführt; bei einer erwarteten Zellhäufigkeit >5 wurden *Chi*^*2*^*-Tests* angewandt und bei einer erwarteten Zellhäufigkeit <5 wurde der *Fisher-Exakt-Test *durchgeführt. Zudem wurden bivariate Korrelationsanalysen nach Pearson sowie der t‑Test bei unabhängigen Stichproben durchgeführt. *p*-Werte <0,05 galten als statistisch signifikant.

## Ergebnisse

**Figure 1:**
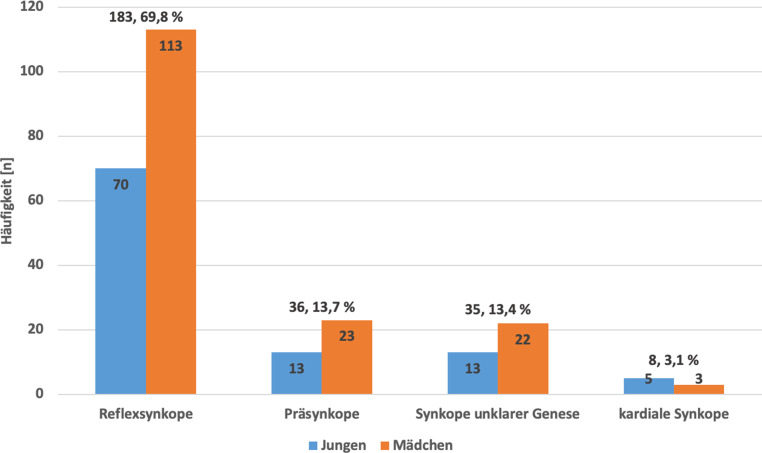

Im erhobenen Zeitraum 01/2015 bis 12/2017 kam es zu 348 Erstvorstellungen wegen „Synkope und Kollaps“ (ICD-10 Code R55); 86 Fälle (24,7 %) wurden von der Datenauswertung ausgeschlossen; 69 Patienten (19,8 %) erfüllten nicht die Einschlusskriterien: 28 der ausgeschlossenen Patienten waren bereits vor 2015 mit einer Synkope am UKS vorstellig und fielen damit nicht in den festgelegten Erhebungszeitraum, bei 18 Patienten ließen sich andere Behandlungsgründe als eine Synkope ermitteln, 17 Patienten hatten keinen Anhalt auf eine stattgehabte Synkope/Präsynkope, und bei 6 Patienten waren nicht ausreichend Daten in der elektronischen Datenbank SAP verfügbar. Bei 17 Patienten (4,9 %) konnten Differenzialdiagnosen der Synkope als Ursache des transienten Bewusstseinsverlustes ermittelt werden: 11 Patienten (64,7 %) mit Epilepsie, 3 (17,7 %) mit kurzzeitigem Bewusstseinsverlust infolge einer Intoxikation, 2 (11,8 %) mit psychogener Pseudosynkope und 1 Patient (5,9 %) mit Hypoglykämie-bedingtem Bewusstseinsverlust. Resultierend wurden 262 Patienten (75,3 %) mit Synkope in der Endauswertung berücksichtigt: 161 Patienten (61,5 %) waren weiblich, 101 Patienten (38,5 %) männlich; das mittlere Alter lag bei 12,5 ± 3,9 Jahre (Altersspanne 1 bis 18 Jahre). Wie in Abb. 1 dargestellt, war die Reflexsynkope (*n* = 183, 69,8 %) die am häufigsten aufgetretene Synkopenentität in unserer Kohorte.

Es wurden 164 Patienten (62,6 %) stationär und 98 (37,4 %) ambulant wegen Synkope behandelt (stationär 4,0 ± 1,4; 0 bis 8 Untersuchungen pro Patient vs. ambulant 1,1 ± 1,3; 0 bis 4 Untersuchungen pro Patient) (Tab. 1). Die mittlere stationäre Aufenthaltsdauer betrug 2,9 Tage ±1,8 (Spannweite 1 bis 19 Tage). Bei 53 Patienten (20,2 %) handelte es sich bei der Synkope um ein Erstereignis, bei 101 Patienten (38,5 %) um eine Rezidivsynkope. Bei den verbleibenden 108 Patienten (41,2 %) ließ sich retrograd nicht eruieren, ob es sich um ein Erstereignis oder Rezidiv handelte.

**Table 1:** 

Diagnostikum	Stationär (*n* = 164)	Ambulant (*n* = 98)
Elektrokardiographie (*n* = 205)	148 (90,2 %)	57 (58,2 %)
Echokardiographie (*n* = 182)	136 (82,9 %)	46(46,9 %)
Labor (*n* = 177)	159 (96,9 %)	18 (18,4 %)
Elektroenzephalografie (*n* = 134)	114 (69,5 %)	20 (20,4 %)
Langzeit-Elektrokardiographie (*n* = 93)	57 (34,8 %)	36 (36,7 %)
Kranielle Magnetresonanztomographie (*n* = 20)	18 (10,9 %)	2 (2,0 %)
Ergometrie (*n* = 15)	10 (6,1 %)	5 (5,1 %)
Blutzuckertagesprofil (*n* = 11)	11 (6,7 %)	0
Langzeitblutdruckmessung (*n* = 6)	3 (1,8 %)	3 (3,1 %)
Schellong-Test (*n* = 3)	3 (1,8 %)	0

Anamnestisch wurden bei 196 Patienten (74,8 %) Vorerkrankungen abgefragt; bei 69 (35,2 %) dieser Patienten ließen sich Vorerkrankungen finden (Tab. 2). Eine Medikamentenanamnese wurde bei 161 Patienten (61,5 %) erhoben; bei 40 Patienten (24,8 %) erfolgte eine Medikamenteneinnahme (Tab. 2). Die Familienanamnese wurde bei 78 Patienten (29,8 %) erhoben und erbrachte bei 24 Patienten (30,8 %) eine familiäre Vorerkrankung. Davon waren 13 (54,2 %) neurologischer und 11 (45,8 %) kardiovaskulärer Genese. Hinweise auf einen plötzlichen Herztod vor dem 40. Lebensjahr fanden sich in keiner Anamnese.

**Table 2:** 

Parameter	Unterkategorie	Anzahl *n* (%)
Vorerkrankungen (*n* = 69)	Kardial	18 (26,1 %)
	Pulmonal	13 (18,8 %)
	Kinder- und jugendpsychiatrisch	12 (17,4 %)
	Neurologisch	11 (15,9 %)
	Hämatologisch	7 (10,1 %)
	Metabolisch	5 (7,2 %)
	Sonstige	3 (4,3 %)
Medikamente (*n* = 40)	Hormonelle Kontrazeption	16 (40,0 %)
	Neurologisch/psychiatrisch	8 (20,0 %)
	Kardiovaskulär	6 (15,0 %)
	Metabolisch	4 (10,0 %)
	Hämatologisch	3 (7,5 %)
	Pulmonal	2 (5,0 %)
	Sonstige	1 (2,5 %)
Prodromi (*n* = 138)	Vertigo	65 (47,1 %)
	Schwarz werden vor Augen	37 (26,8 %)
	Nausea	22 (15,9 %)
	Zephalgie	6 (4,3 %)
	Sonstige	4 (2,9 %)
	Augenflimmern	2 (1,4 %)
	Hyperhidrose	2 (1,4 %)
Trigger (*n* = 97)	Orthostatischer Lagewechsel	25 (25,8 %)
	Schmerz	15 (15,5 %)
	Unangenehmer Anblick/Geruch	15 (15,5 %)
	Nach körperlicher Belastung	14 (14,4 %)
	Langes Stehen	11 (11,3 %)
	Emotionale Belastung	9 (9,3 %)
	Wärme	4 (4,1 %)
	Postprandial	4 (4,1 %)

Eine vollständige Anamnese (Basisdiagnostik I), bestehend aus Vorerkrankungen, Medikamentenanamnese und Familienanamnese, wurde bei insgesamt 46 Patienten (17,6 %) erhoben. Bei diesen Patienten wurden gemittelt 4,1 ± 1,5 Untersuchungen (0 bis 7 Untersuchungen) pro Patient durchgeführt, während es bei Patienten mit unvollständig erhobener Anamnese (*n* = 216; 82,4 %) gemittelt 3,0 ± 1,7 Untersuchungen (0 bis 8 Untersuchungen) pro Patient waren (*p* < 0,01). Die vollständige Erhebung der Anamnese (Basisdiagnostik I) führte nicht zu einer besseren Zuordenbarkeit der Synkopenentität (*p* = 0,15).

Prodromi vor dem Auftreten der Synkope fanden sich bei 138 Patienten (52,7 %) (Tab. 2). Trigger für eine Synkope fanden sich anamnestisch bei 97 Patienten (37,0 %) (Tab. 2). Zusätzlich bestand bei 43 Patienten (16,4 %) zum Zeitpunkt des Auftretens der Synkope ein Infekt, und bei 70 Patienten (26,7 %) fanden sich in der Anamnese zusätzlich Hinweise darauf, dass am Tag der Synkope wenig gegessen/getrunken wurde.

Bei 42 Patienten (16,0 %) ließen sich „red flags“ in der Anamnese erheben. Bei 17 Patienten (40,5 %) trat die Synkope während körperlicher Belastung auf, 13 Patienten (31,0 %) hatten keine Prodromi vor der Synkope, bei 6 Patienten (14,3 %) folgte die Synkope auf Brustschmerz oder Palpitationen, und 3 Patienten (7,1 %) erlitten die Synkope im Liegen. Zudem wurden bei 3 Patienten (7,1 %) mehrere „red flags“ gleichzeitig gefunden. Ein statistisch signifikanter Zusammenhang zwischen dem Vorliegen von „red flags“ in der Anamnese und dem Auftreten kardialer Synkopen konnte nicht gefunden werden (*p* = 0,61).

Auch ließ sich kein signifikanter Zusammenhang zwischen dem Vorliegen von „red flags“ und vermehrt durchgeführten apparativen Untersuchungen im Vergleich zu Patienten ohne „red flags“ finden (*p* = 0,53); aber beim Vorliegen von „red flags“ fand sich signifikant häufiger mindestens eine Pathologie in der apparativen Diagnostik (*p* = 0,048); 42 Patienten (16,0 %) mit „red flags“ erhielten 142/846 Untersuchungen (3,4 ± 1,8 Untersuchungen pro Patient); 220 Patienten (84,0 %) ohne „red flags“ erhielten 704/846 apparative Untersuchungen (3,2 ± 1,7 Untersuchungen pro Patient).

Die mit Abstand häufigsten apparativen Untersuchungen stellten das EKG (*n* = 205, 78,2 %) sowie die TTE (*n* = 182, 69,5 %) dar; hierbei erbrachten das EKG (*n* = 19/846,  2,2 %) und das LZ-EKG (*n* = 19/846, 2,2 %) die meisten pathologischen Befunde (Tab. 3).

**Table 3:** 

Diagnostik	Anzahl durchgeführter Untersuchungen (*n* = 846)	Anzahl pathologischer Befunde (*n* = 58)
*Elektrokardiographie*	205 (78,2 %)	19 (9,3 %)
*Echokardiographie*	182 (69,5 %)	1 (0,5 %)
*Labor*	177 (67,6 %)	2 (1,1 %)
*Elektroenzephalographie*	134 (51,1 %)	12 (8,9 %)
*Langzeitelektrokardiographie*	93 (35,5 %)	19 (20,4 %)
*Kranielle Magnetresonanztomographie*	20 (7,6 %)	4 (20,0 %)
*Ergometrie*	15 (5,7 %)	0 (0,0 %)
*Blutzuckertagesprofil*	11 (4,2 %)	0 (0,0 %)
*Langzeitblutdruckmessung*	6 (2,3 %)	0 (0,0 %)
*Schellong-Test*	3 (1,1 %)	1 (33,3 %)

Patienten mit kardialer Synkope wurden gemittelt 3,9 ± 1,1 apparativen Untersuchungen (3 bis 6 Untersuchungen) pro Patient zugeführt, und bei diesen Patienten fanden sich auch die meisten pathologischen Befunde (1,4 ± 0,7; 1 bis 3 Pathologien pro Patient). Im Gegensatz dazu wurden bei Patienten mit Präsynkope am wenigsten Untersuchungen durchgeführt (2,5 ± 1,6; 0 bis 6 Untersuchungen pro Patient) und gemittelt 0,2 ± 0,5 pathologische Befunde (0 bis 2 Pathologien) pro Patient erhoben. Die Anzahl der durchgeführten apparativen Untersuchungen pro Patient und die Anzahl der dabei erhobenen pathologischen Befunde zeigten einen signifikanten Zusammenhang (*p* < 0,001).

Bezogen auf den Einsatz der Leitlinie bei den insgesamt 262 eingeschlossenen Patienten zeigte sich, dass 74/262 Patienten (28,2 %) mit durchgeführter Basisdiagnostik II + III, bei denen weiterführende Diagnostik indiziert war, diese auch erhielten; 188/262 Patienten (71,8 %) wurden nicht adäquat diagnostiziert; 77/188 (41,0 %) der Patienten erhielten keine ausreichende Basisdiagnostik II + III, während bei 111/188 Patienten (59,0 %), bei denen die Basisdiagnostik II + III ausreichend zur Diagnosestellung gewesen wäre, zusätzliche nicht indizierte apparative Untersuchungen durchgeführt wurden. Bezogen auf die häufigsten weiterführenden apparativen Untersuchungen wurden 184 unnötige Untersuchungen durchgeführt (82 TTEs, 69 EEGs und 33 LZ-EKGs). Eine vollständige Basisdiagnostik II + III wurde bei insgesamt 185 Patienten (70,6 %) durchgeführt. Diese führte aber nicht zu einer besseren Zuordenbarkeit der Synkopenentität (*p* = 0,23).

**Figure 2:**
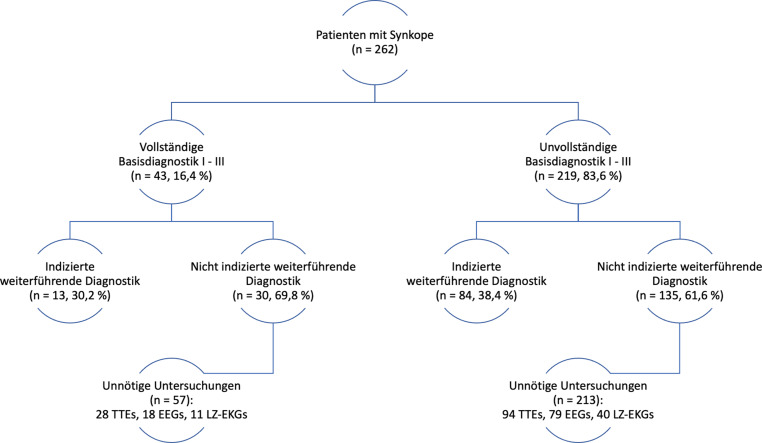

Eine vollständige Basisdiagnostik (I–III) wurde bei insgesamt 43/262 Patienten (16,4 %) erhoben; 219/262 Patienten (83,6 %) erhielten keine ausreichende Basisdiagnostik I–III (Abb. 2). Bezogen auf die insgesamt 409 durchgeführten Untersuchungen in dem Patientenkollektiv (davon 182 TTEs, 134 EEGs und 93 LZ-EKGs) waren 270 dieser Untersuchungen (66,0 %) nicht indiziert.

## Diskussion

In dieser retrospektiven Studie konnten wir zeigen, dass das diagnostische Vorgehen bei Kindern und Jugendlichen mit Synkope in unserer Klinik in vielen Fällen nicht gemäß der S2k-Leitlinie [8] der DGPK erfolgte.

Bezogen auf die Synkopenentität handelte es sich in unserer Population, wie auch in anderen Studien bereits beschrieben, am häufigsten um gutartige Reflexsynkopen [19, 22, 28]. Trotz der Ausschlusskriterien und Ermittlung der wichtigsten Differenzialdiagnosen blieb in 13,4 % die Genese der Synkope unklar, was am ehesten einer unvollständigen Dokumentation geschuldet war. Verschiedene Arbeiten zeigen, dass die Implementierung der pädiatrischen Synkopenleitlinie zu einer Reduktion der Anzahl undefinierter Synkopen führte [11, 28].

Im Einklang mit vorangegangenen Studien (2–6 %) [12, 32] fanden sich in unserer Untersuchung in 3,1 % der Fälle kardiale Synkopen. Patienten mit „red flags“ in der Anamnese wird ein erhöhtes Risiko für kardiale Synkopen zugeschrieben [23, 27, 32], weshalb diese Patienten einer weiterführenden Diagnostik zugeführt werden sollten. Allerdings ließ sich in unserer Erhebung kein statistischer Zusammenhang zwischen dem Vorhandensein von „red flags“ und dem Auftreten einer kardialen Synkope finden (*p* = 0,61).

Bei mehr als jedem dritten Patienten (38,5 %) kam es in unserer Erhebung zu einer Rezidivsynkope. In der Literatur finden sich hierzu sehr variable Angaben. Während Colman et al. ein Rezidivrisiko für etwa jeden zweiten Patienten mit Reflexsynkope beschreiben [4], findet sich bei Massin [22] nur bei 23,8 % der Synkopen ein Rezidiv. Wir konnten keinen Zusammenhang zwischen der Entität der Synkope und der Häufigkeit eines Rezidivs feststellen (*p* = 0,87).

Interessanterweise wurden bei Patienten mit vollständiger Anamnese statistisch mehr apparative Untersuchungen (4,1 ± 1,5; 0 bis 7 Untersuchungen pro Patient) durchgeführt als bei Patienten, bei denen dies nicht erfolgte (3,0 ± 1,7; 0 bis 8 Untersuchungen pro Patient). Dies könnte möglicherweise auf einen unterschiedlichen diagnostischen Ansatz der behandelnden Ärzte zurückzuführen sein und ggf. auf Unterschieden im Ausbildungsstand basieren (unerfahrenere Ärzte: ausführlichere Anamnese und mehr apparative Diagnostik vs. erfahrenere Ärzte: fokussierte Anamnese und weniger apparative Diagnostik).

In unserer Studie wurde bei nahezu allen Patienten (98,1 %) eine körperliche Untersuchung durchgeführt, aber nur bei einem geringen Anteil (28,0 %) fanden sich hierbei pathologische Befunde. Johnson et al. [20] fanden bei 617 eingeschlossenen Patienten nur bei 4 % auffällige Befunde in der körperlichen Untersuchung. In der Studie von Massin [22] waren fast alle Befunde der körperlichen Untersuchung bei den 226 eingeschlossenen Patienten Normalbefunde; lediglich bei 3,5 % der Patienten fand sich entweder ein neurologisches Defizit oder ein pathologischer Auskultationsbefund des Herzens [22]. Es ist zu vermuten, dass der körperliche Untersuchungsbefund nach stattgehabter Synkope in den allermeisten Fällen unauffällig oder unspezifisch ist, es sei denn, es liegt eine verursachende Grunderkrankung vor.

Die Durchführung einer Ruheblutdruckmessung erfolgte bei einem Großteil unserer Patienten (85,5 %). Rückschlüsse der Messergebnisse auf eine orthostatische Hypotension bei den Patienten mit Synkope ließen sich allerdings nicht ziehen.

Das 12-Kanal-EKG war in unserer Studie das am Häufigsten angewandte apparative Diagnostikum (78,2 %) und wies mit 9,3 % die höchste Rate an pathologischen Befunden auf. Alle Patienten mit kardialer Synkope erhielten ein 12-Kanal-EKG (3,1 %). In einer Studie von Johnson konnten in der Zusammenschau von Anamnese, körperlicher Untersuchung und EKG alle Patienten mit schwerwiegender Ursache der Synkope ermittelt werden [20]. Dieses Ergebnis steht in einem gewissen Widerspruch zu unserem Ergebnis, da in unserer Untersuchung Patienten mit kardialer Synkope allein durch das LZ-EKG detektiert wurden. Allerdings kann je nach Ausbildungsstand der behandelnden Ärzte die Interpretation des EKGs fehlerbehaftet sein, weshalb die Befundung durch einen Kinderkardiologen sinnvoll ist [15]. Guse und Raucci beschreiben einen Anstieg der Durchführung eines EKGs nach Implementierung einer Synkopenleitlinie [16, 28]. Die Wichtigkeit der Ableitung eines 12-Kanal-EKGs als Screeningmethode, insbesondere für eine potenzielle arrhythmogene kardiale Synkope, wird untermauert durch Ergebnisse einer Studie aus China [32]. Hier hatten 29/31 Patienten mit kardialer Synkope ein auffälliges EKG im Gegensatz zu 5/55 Patienten mit Reflexsynkope [32].

Die Durchführung einer Echokardiographie ist gemäß DGPK-Leitlinie nicht notwendig, wenn durch die initial durchgeführte Diagnostik sicher auf eine Reflexsynkope geschlossen werden kann [8]. Nur bei ca. 0,5–2 % der Synkopen finden sich pathologische Befunde, die potenziell über die Echokardiographie erhoben werden können [14, 22, 25, 29, 31]. In unserer Studie war die Echokardiographie mit 69,5 % die zweithäufigste eingesetzte apparative Untersuchung, wobei diese in 45,1 % der Fälle nicht indiziert war. Eine Vermutung für den hohen Einsatz dieser Untersuchung ist die geringe Hemmschwelle zur Durchführung der Untersuchung aufgrund der einfachen Verfügbarkeit in unserer Kinderklinik.

Auch Laboruntersuchungen (Blutbild, klinische Chemie sowie Blutgasanalysen) wurde bei unseren Patienten großzügig eingesetzt (67,6 %), erbrachten aber nahezu keine auffälligen Ergebnisse (1,1 %) – vergleichbar mit den Ergebnissen anderer Arbeiten [16, 28]. Eine Laboruntersuchung sollte bei unklarem Bewusstseinsverlust oder nichtsynkopalem Bewusstseinsverlust erfolgen und dient dann z. B. dem Ausschluss einer Hypoglykämie, relevanter Elektrolytstörungen oder einer Anämie.

Der Einsatz des EEGs erwies sich in unserer Untersuchung als häufig (51,1 %), aber wenig zielführend. Generell wird empfohlen, bei unklarem oder nichtsynkopalem Bewusstseinsverlust sowie bei Hinweisen auf ein epileptisches Geschehen ein EEG durchzuführen [8]. Aufgrund der geringen Ausbeute an pathologischen Befunden ist das EEG allerdings nicht sinnvoll in der Standardabklärung von Synkopen [1, 5, 6, 29]. Bei Raucci war ein Rückgang des Einsatzes eines EEG nach Implementierung der Synkopenleitlinie zu verzeichnen [28].

Das LZ-EKG ist indiziert bei einem begründeten Verdacht auf arrhythmogene Ursachen und bei Patienten mit häufigen Rezidiven innerhalb weniger Tage. In unserer Studie wurde es bei 35,5 % der Patienten durchgeführt und zeigte in 20,4 % pathologische Befunde. Alle 8 Patienten mit kardialer Synkope erhielten ein LZ-EKG und wurden durch die dort erhobenen Befunde diagnostiziert; bei 35,5 % der durchgeführten LZ-EKGs bestand hierfür aber gemäß DGPK-Leitlinie keine Indikation. Wiederum bei Raucci fand sich eine Reduktion des Einsatzes der LZ-EKG-Untersuchung nach Implementierung der Synkopenleitlinie (Präinterventionsgruppe: 9,1 % vs. Postinterventionsgruppe: 3,3 %) [28].

### Zusammenfassung

Zusammenfassend sahen wir in unserer Studie eine erhebliche Differenz zwischen der zum Zeitpunkt der Untersuchung gültigen aktuellen Handlungsempfehlung [8, 9] und klinischer Umsetzung bei Patienten mit Synkope. Die Abweichung bestand im Wesentlichen darin, dass grundlegend empfohlene Untersuchungen (Basisdiagnostik I–III) nicht vollständig durchgeführt, andererseits aber nicht notwendige, weiterführende Diagnostik ohne Vorhandensein einer vollständigen Basisdiagnostik (I–III) durchgeführt wurde. Dadurch kam es zu einer unnötigen Überdiagnostik, die auch zu einer Verunsicherung von Patienten und Angehörigen führen kann.

Eine Ursache für die Differenz zwischen den bestehenden Empfehlungen und dem Vorgehen im klinischen Alltag könnte ein mangelndes Bewusstsein der behandelnden Ärzte für das Vorliegen der DGPK-Leitlinie sein. Allein das Vorliegen von Leitlinien führt sicherlich nicht zu einer Änderung der klinischen Praxis. Dies bedarf vielmehr der Schulung des ärztlichen Personals und der Sensibilisierung, Handlungsempfehlungen im klinischen Alltag ein- und umzusetzen. Um eine optimale Umsetzung von nationalen und internationalen Leitlinien in den Klinikalltag zu realisieren, ist es zudem erforderlich, Leitlinien in klinikinterne SOPs einzuarbeiten und regelmäßige Schulungen des Personals diesbezüglich vorzunehmen. Basierend auf den Ergebnissen unserer Studie, ist es Ziel unserer Klinik, hierfür, aber auch in anderen Bereichen (z. B. Vorgehen bei Kindern und Jugendlichen mit Thoraxschmerzen) interne SOPs zu erstellen und regelmäßige Schulungen zu implementieren. Für Kinder mit Synkopen muss es das Ziel sein, eine vollständige Basisdiagnostik (Anamnese, körperliche Untersuchung und 12-Kanal-EKG) durchzuführen und eine weitergehende Diagnostik – so indiziert – anzuschließen; gleichzeitig ist eine unnötige und nicht indizierte Überdiagnostik zu vermeiden. Die Anamnese als Bestandteil der Basisdiagnostik ist dabei von besonderer Wichtigkeit. Die Implementierung der S2k-Leitlinie „Synkope im Kindes- und Jugendalter“ der DGPK [8], die 02/2020 noch mal aktualisiert wurde [7], stellt hierfür ein geeignetes Instrumentarium dar.

## Abkürzungen

BZTP: Blutzuckertagesprofil
cMRT: Kranielle Magnetresonanztomographie,
DGPK: Deutsche Gesellschaft für Pädiatrische Kardiologie und Angeborene Herzfehler e. V.,
EEG: Elektroenzephalographie
EKG: Elektrokardiographie
LZ-EKG: Langzeitelektrokardiographie
LZ-RR: Langzeitblutdruckmessung
TTE: Transthorakale Echokardiographie
UKS: Uniklinikum des Saarlandes
